# The genome sequence of the lime hawk-moth, *Mimas tiliae *(Linnaeus, 1758)

**DOI:** 10.12688/wellcomeopenres.17485.1

**Published:** 2021-12-21

**Authors:** Douglas Boyes, Peter W.H. Holland, University of Oxford and Wytham Woods Genome Acquisition Lab, Darwin Tree of Life Barcoding collective, Wellcome Sanger Institute Tree of Life programme, Wellcome Sanger Institute Scientific Operations: DNA Pipelines collective, Tree of Life Core Informatics collective, Darwin Tree of Life Consortium

**Affiliations:** 1 UK Centre for Ecology and Hydrology, Wallingford, Oxfordshire, UK; 2 Department of Zoology, University of Oxford, Oxford, UK

**Keywords:** Mimas tiliae, lime hawk-moth, genome sequence, chromosomal, Lepidoptera

## Abstract

We present a genome assembly from an individual male *Mimas tiliae *(the lime hawk-moth; Arthropoda; Insecta; Lepidoptera; Sphingidae). The genome sequence is 478 megabases in span. The complete assembly is scaffolded into 29 chromosomal pseudomolecules, with the Z sex chromosome assembled.

## Species taxonomy

Eukaryota; Metazoa; Ecdysozoa; Arthropoda; Hexapoda; Insecta; Pterygota; Neoptera; Endopterygota; Lepidoptera; Glossata; Ditrysia; Bombycoidea; Sphingidae; Smerinthinae; Smerinthini; Mimas; *Mimas tiliae* (Linnaeus, 1758) (NCBI:txid522848).

## Background

*Mimas tiliae* (lime hawk-moth) is characterised by scalloped edges to the forewing, along with bold green and buff markings, which are thought to disrupt object perception by predators through 'disruptive coloration' ( Stevens *et al*., 2006). The wing markings also show ‘edge enhanced colouration’ which may also slow object recognition by a predator ( Sharman *et al*., 2018). *Mimas tiliae* is common throughout southern England, particularly London, and has spread further north in recent years, with one record of breeding as far north as Glasgow, Scotland ( Payne, 2020). The species can be found in woodland and in suburban habitats, and flies in May and June. The genome of *M. tiliae* was sequenced as part of the Darwin Tree of Life Project, a collaborative effort to sequence all of the named eukaryotic species in the Atlantic Archipelago of Britain and Ireland. Here we present a chromosomally complete genome sequence for *M. tiliae*, based on one male specimen from Wytham Woods, Oxfordshire, UK.

## Genome sequence report

**Figure 1.**
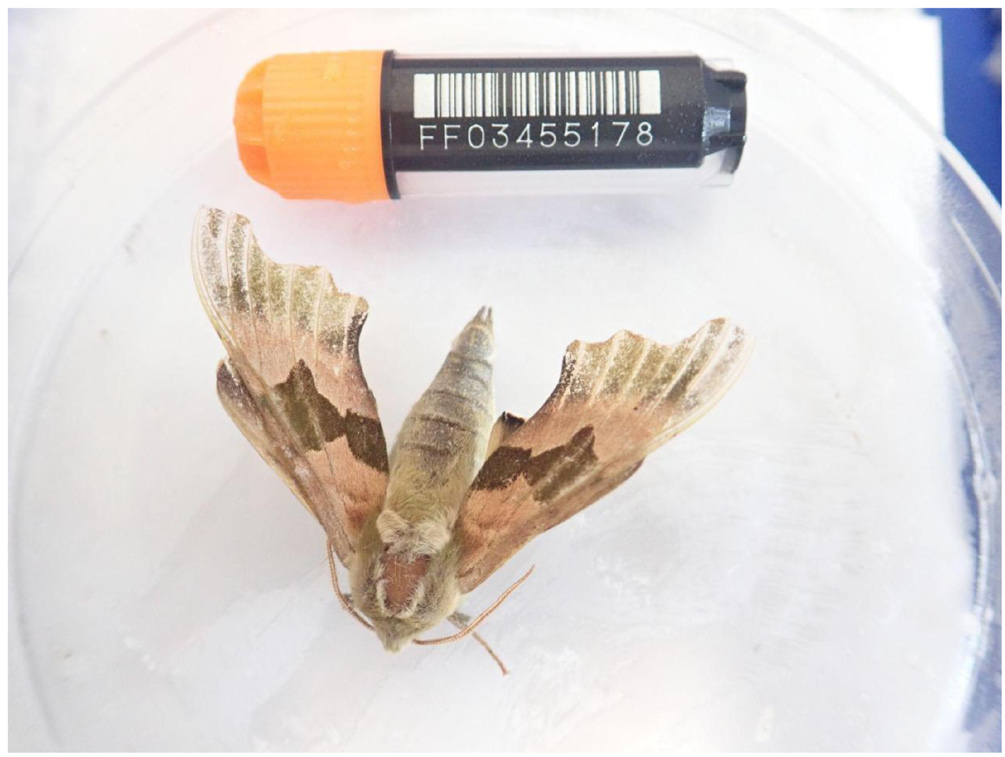
Image of the ilMimTili1 specimen taken during preservation and processing.

The genome was sequenced from a single male *M. tiliae* ( Figure 1) collected from Wytham Woods, Oxfordshire, UK (latitude 51.768, longitude -1.337). A total of 37-fold coverage in Pacific Biosciences single-molecule long reads and 80-fold coverage in 10X Genomics read clouds were generated. Primary assembly contigs were scaffolded with chromosome conformation Hi-C data. Manual assembly curation corrected 10 missing/misjoins and removed 2 haplotypic duplications, reducing the assembly length by 0.05% and the scaffold number by 21.62%.

**Figure 2.**
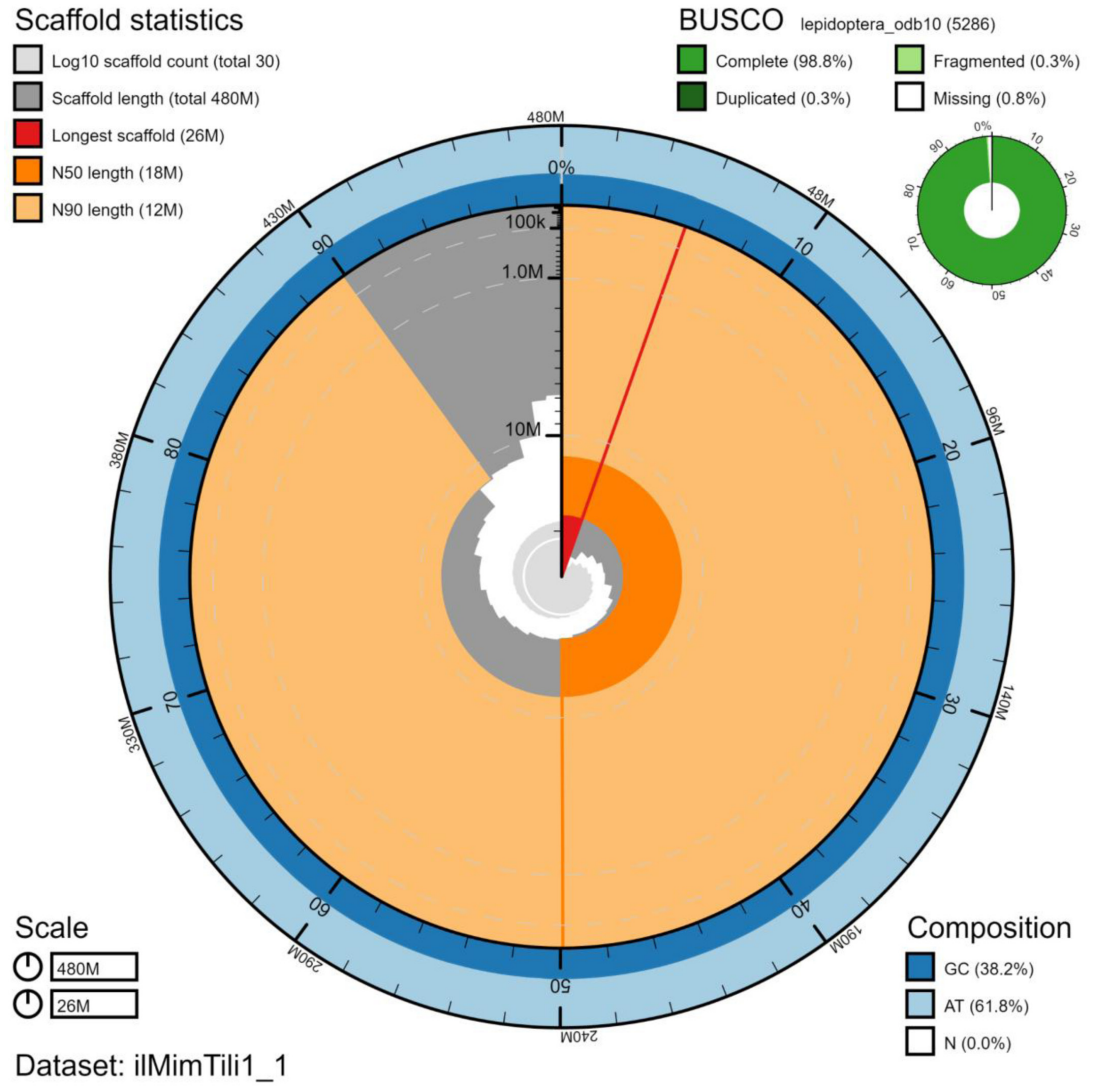
Genome assembly of *Mimas tiliae*, ilMimTili1.1: metrics. The BlobToolKit Snailplot shows N50 metrics and BUSCO gene completeness. The main plot is divided into 1,000 size-ordered bins around the circumference with each bin representing 0.1% of the 477,981,037 bp assembly. The distribution of chromosome lengths is shown in dark grey with the plot radius scaled to the longest chromosome present in the assembly (25,978,239 bp, shown in red). Orange and pale-orange arcs show the N50 and N90 chromosome lengths (17,899,878 and 11,870,120 bp), respectively. The pale grey spiral shows the cumulative chromosome count on a log scale with white scale lines showing successive orders of magnitude. The blue and pale-blue area around the outside of the plot shows the distribution of GC, AT and N percentages in the same bins as the inner plot. A summary of complete, fragmented, duplicated and missing BUSCO genes in the lepidoptera_odb10 set is shown in the top right. An interactive version of this figure is available at https://blobtoolkit.genomehubs.org/view/Mimas%20tiliae/dataset/ilMimTili1_1/snail.

The final assembly has a total length of 478 Mb in 29 sequence scaffolds with a scaffold N50 of 18 Mb ( Table 1). Of the assembly sequence, 100% was assigned to 29 chromosomal-level scaffolds, representing 28 autosomes (numbered by sequence length), and the Z sex chromosome ( Figure 2– Figure 5; Table 2). The assembly has a BUSCO v5.1.2 ( Simão *et al*., 2015) completeness of 98.8% (single 98.5%, duplicated 0.3%) using the lepidoptera_odb10 reference set. While not fully phased, the assembly deposited is of one haplotype. Contigs corresponding to the second haplotype have also been deposited.

**Table 1.** Genome data for *Mimas tiliae*, ilMimTili1.1.

*Project accession data*	
Assembly identifier	ilMimTili1.1
Species	*Mimas tiliae*
Specimen	ilMimTili1
NCBI taxonomy ID	NCBI:txid522848
BioProject	PRJEB43536
BioSample ID	SAMEA7520521
Isolate information	Male, head/thorax/abdomen
*Raw data accessions*	
PacificBiosciences SEQUEL I	ERR6394585
10X Genomics Illumina	ERR6054525-ERR6054528
Hi-C Illumina	ERR6054524
*Genome assembly*	
Assembly accession	GCA_905332985.1
*Accession of alternate haplotype*	GCA_905333085.1
Span (Mb)	478
Number of contigs	40
Contig N50 length (Mb)	17.8
Number of scaffolds	29
Scaffold N50 length (Mb)	17.9
Longest scaffold (Mb)	26.0
BUSCO * genome score	C:98.8%[S:98.5%,D:0.3%], F:0.3%,M:0.8%,n:5286

*BUSCO scores based on the lepidoptera_odb10 BUSCO set using v5.1.2. C= complete [S= single copy, D=duplicated], F=fragmented, M=missing, n=number of orthologues in comparison. A full set of BUSCO scores is available at https://blobtoolkit.genomehubs.org/view/Mimas%20tiliae/dataset/ilMimTili1_1/busco.

**Figure 3.**
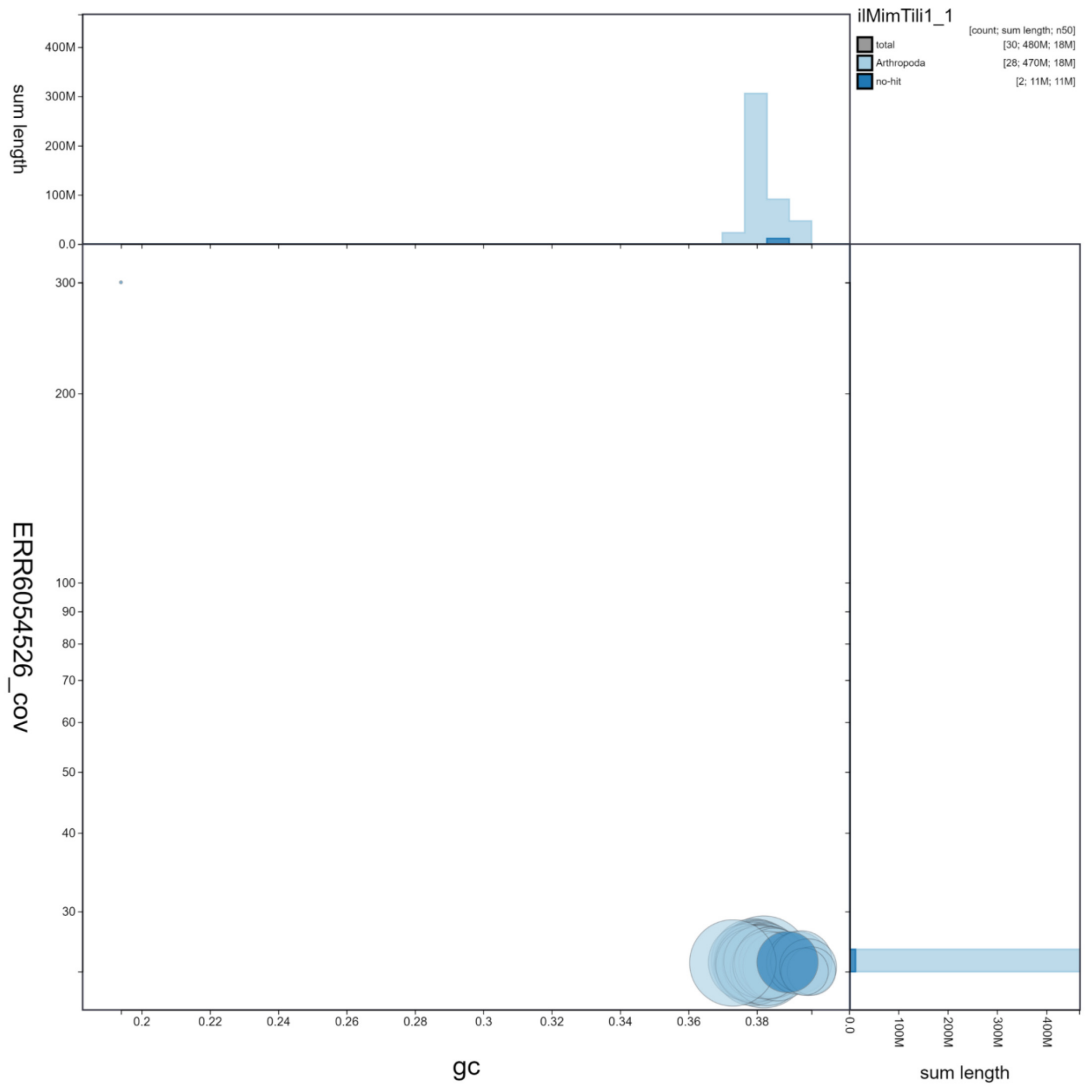
Genome assembly of *Mimas tiliae*, ilMimTili1.1: GC coverage. BlobToolKit GC-coverage plot. Scaffolds are coloured by phylum. Circles are sized in proportion to scaffold length. Histograms show the distribution of scaffold length sum along each axis. An interactive version of this figure is available at https://blobtoolkit.genomehubs.org/view/Mimas%20tiliae/dataset/ilMimTili1_1/blob.

**Figure 4.**
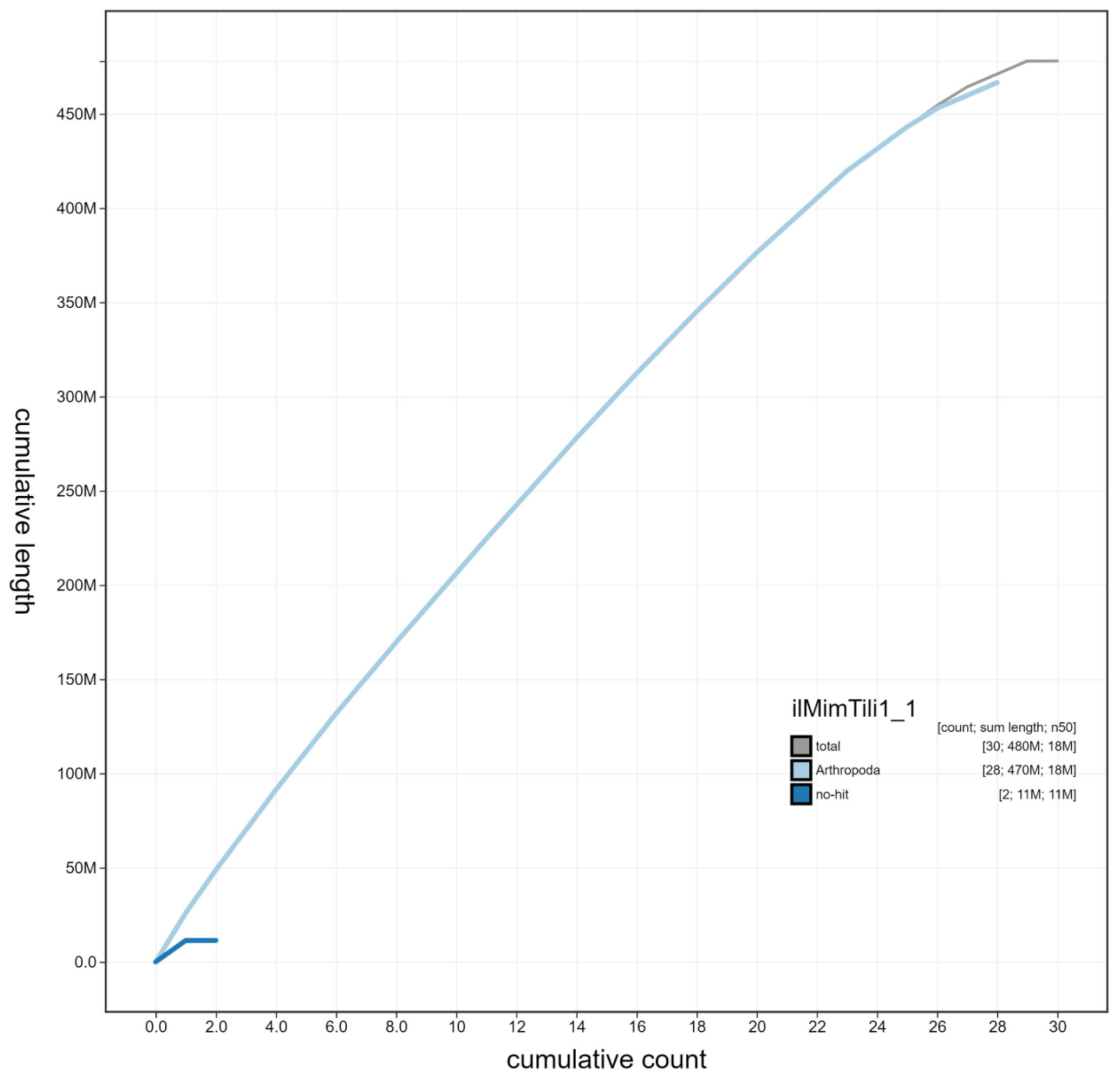
Genome assembly of *Mimas tiliae*, ilMimTili1.1: cumulative sequence. BlobToolKit cumulative sequence plot. The grey line shows cumulative length for all scaffolds. Coloured lines show cumulative lengths of scaffolds assigned to each phylum using the buscogenes taxrule. An interactive version of this figure is available at https://blobtoolkit.genomehubs.org/view/Mimas%20tiliae/dataset/ilMimTili1_1/cumulative.

**Figure 5.**
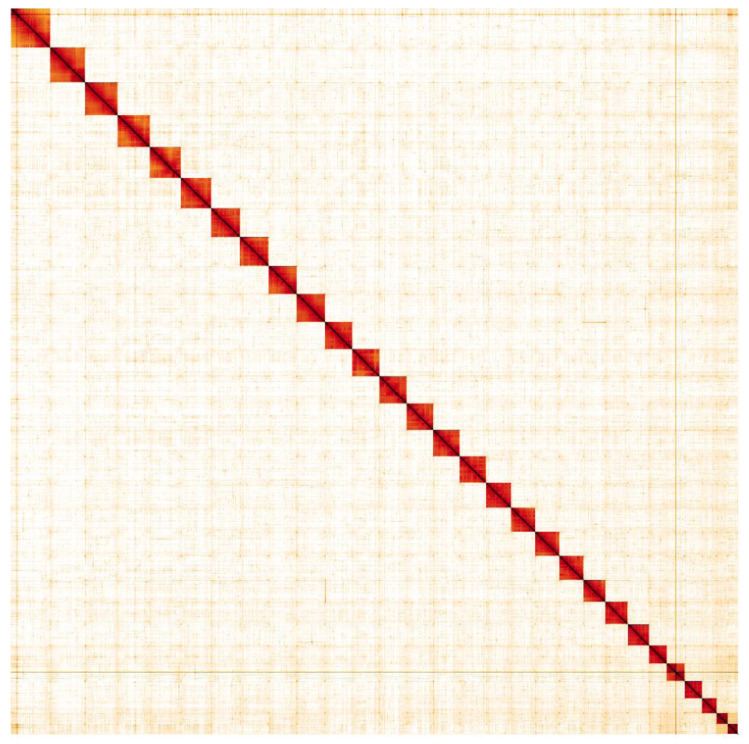
Genome assembly of *Mimas tiliae*, ilMimTili1.1: Hi-C contact map. Hi-C contact map of the ilMimTili1.1 assembly, visualised in HiGlass. Chromosomes are given in order of size from left to right and top to bottom.

**Table 2.** Chromosomal pseudomolecules in the genome assembly of *Mimas tiliae*, ilMimTili1.1.

ENA accession	Chromosome	Size (Mb)	GC%
HG995238.1	1	25.98	38.2
HG995240.1	2	21.59	37.9
HG995241.1	3	20.90	38
HG995242.1	4	20.30	38
HG995243.1	5	20.14	38
HG995244.1	6	19.20	37.9
HG995245.1	7	18.59	37.9
HG995246.1	8	18.42	38
HG995247.1	9	18.34	38.2
HG995248.1	10	18.23	38.1
HG995249.1	11	17.90	38.1
HG995250.1	12	17.80	37.7
HG995251.1	13	17.67	37.9
HG995252.1	14	17.29	38.1
HG995253.1	15	17.06	37.9
HG995254.1	16	16.43	38.3
HG995255.1	17	16.25	38.1
HG995256.1	18	15.70	38.4
HG995257.1	19	15.64	38.4
HG995258.1	20	14.79	38.5
HG995259.1	21	14.46	38.6
HG995260.1	22	14.12	38.3
HG995261.1	23	11.87	39.2
HG995262.1	24	11.51	39.3
HG995263.1	25	11.37	38.9
HG995264.1	26	9.77	39.5
HG995265.1	27	7.02	39.6
HG995266.1	28	6.71	39.4
HG995239.1	Z	22.90	37.3
HG995267.1	MT	0.02	19.7

## Methods

### Sample acquisition and DNA extraction

A single male *M. tiliae* (ilMimTili1) was collected from Wytham Woods, Oxfordshire, UK (latitude 51.768, longitude -1.337) by Douglas Boyes, UKCEH, using a light trap. The specimen was identified by the same individual and preserved on dry ice.

DNA was extracted at the Tree of Life laboratory, Wellcome Sanger Institute. The ilMimTili1 sample was weighed and dissected on dry ice with tissue set aside for Hi-C sequencing. Abdomen tissue was disrupted using a Nippi Powermasher fitted with a BioMasher pestle. Fragment size analysis of 0.01-0.5 ng of DNA was then performed using an Agilent FemtoPulse. High molecular weight (HMW) DNA was extracted using the Qiagen MagAttract HMW DNA extraction kit. Low molecular weight DNA was removed from a 200-ng aliquot of extracted DNA using 0.8X AMpure XP purification kit prior to 10X Chromium sequencing; a minimum of 50 ng DNA was submitted for 10X sequencing. HMW DNA was sheared into an average fragment size between 12-20 kb in a Megaruptor 3 system with speed setting 30. Sheared DNA was purified by solid-phase reversible immobilisation using AMPure PB beads with a 1.8X ratio of beads to sample to remove the shorter fragments and concentrate the DNA sample. The concentration of the sheared and purified DNA was assessed using a Nanodrop spectrophotometer and Qubit Fluorometer and Qubit dsDNA High Sensitivity Assay kit. Fragment size distribution was evaluated by running the sample on the FemtoPulse system.

### Sequencing

Pacific Biosciences HiFi circular consensus and 10X Genomics read cloud sequencing libraries were constructed according to the manufacturers’ instructions. Sequencing was performed by the Scientific Operations core at the Wellcome Sanger Institute on Pacific Biosciences SEQUEL II and Illumina HiSeq X instruments. Hi-C data were generated from head/thorax tissue using the Arima Hi-C+ kit and sequenced on HiSeq X.

### Genome assembly

Assembly was carried out with Hifiasm ( Cheng *et al*., 2021); haplotypic duplication was identified and removed with purge_dups ( Guan *et al*., 2020). One round of polishing was performed by aligning 10X Genomics read data to the assembly with longranger align, calling variants with freebayes ( Garrison & Marth, 2012). The assembly was then scaffolded with Hi-C data ( Rao *et al*., 2014) using SALSA2 ( Ghurye *et al*., 2019). The assembly was checked for contamination and corrected using the gEVAL system ( Chow *et al*., 2016) as described previously ( Howe *et al*., 2021). Manual curation ( Howe *et al*., 2021) was performed using gEVAL, HiGlass ( Kerpedjiev *et al*., 2018) and Pretext. The mitochondrial genome was assembled using MitoHiFi ( Uliano-Silva *et al*., 2021) and annotated using MitoFinder ( Allio *et al*., 2020). The genome was analysed and BUSCO scores generated within the BlobToolKit environment ( Challis *et al*., 2020). Table 3 contains a list of all software tool versions used, where appropriate.

**Table 3.** Software tools used.

Software tool	Version	Source
Hifiasm	0.12	Cheng *et al*., 2021
purge_dups	1.2.3	Guan *et al*., 2020
SALSA2	2.2	Ghurye *et al*., 2019
longranger align	2.2.2	https://support.10xgenomics.com/genome-exome/ software/pipelines/latest/advanced/other-pipelines
freebayes	1.3.1-17-gaa2ace8	Garrison & Marth, 2012
MitoHiFi	1.0	https://github.com/marcelauliano/MitoHiFi
gEVAL	N/A	Chow *et al*., 2016
HiGlass	1.11.6	Kerpedjiev *et al*., 2018
PretextView	0.1.x	https://github.com/wtsi-hpag/PretextView
BlobToolKit	2.6.2	Challis *et al*., 2020

### Ethics/compliance issues

The materials that have contributed to this genome note have been supplied by a Darwin Tree of Life Partner. The submission of materials by a Darwin Tree of Life Partner is subject to the Darwin Tree of Life Project Sampling Code of Practice. By agreeing with and signing up to the Sampling Code of Practice, the Darwin Tree of Life Partner agrees they will meet the legal and ethical requirements and standards set out within this document in respect of all samples acquired for, and supplied to, the Darwin Tree of Life Project. Each transfer of samples is further undertaken according to a Research Collaboration Agreement or Material Transfer Agreement entered into by the Darwin Tree of Life Partner, Genome Research Limited (operating as the Wellcome Sanger Institute), and in some circumstances other Darwin Tree of Life collaborators.

## Data availability

European Nucleotide Archive: Mimas tiliae (lime hawk-moth). Accession number PRJEB43536: https://www.ebi.ac.uk/ena/browser/view/PRJEB43536.

The genome sequence is released openly for reuse. The *M. tiliae* genome sequencing initiative is part of the Darwin Tree of Life (DToL) project. All raw sequence data and the assembly have been deposited in INSDC databases. The genome will be annotated and presented through the Ensembl pipeline at the European Bioinformatics Institute. Raw data and assembly accession identifiers are reported in Table 1.

## Author information

Members of the University of Oxford and Wytham Woods Genome Acquisition Lab are listed here: https://doi.org/10.5281/zenodo.5746938.

Members of the Darwin Tree of Life Barcoding collective are listed here: https://doi.org/10.5281/zenodo.5744972.

Members of the Wellcome Sanger Institute Tree of Life programme are listed here: https://doi.org/10.5281/zenodo.5744840.

Members of Wellcome Sanger Institute Scientific Operations: DNA Pipelines collective are listed here: https://doi.org/10.5281/zenodo.5746904.

Members of the Tree of Life Core Informatics collective are listed here: https://doi.org/10.5281/zenodo.5743293.

Members of the Darwin Tree of Life Consortium are listed here: https://doi.org/10.5281/zenodo.5638618.
