# Supplementary material for: The genome sequence of the lime hawk-moth, Mimas tiliae (Linnaeus, 1758)
Source: Wellcome Open Res. 2021 Dec 21;6:357. [Version 1] doi: 10.12688/wellcomeopenres.17485.1 (PMC8844536; doi:10.12688/wellcomeopenres.17485.1)
Supplement: Peer review history

# Peer review: Reviewer response for version 1

**DOI:** 10.21956/wellcomeopenres.19335.r47759

**Published:** 2022-02-14

**Authors:** Winston Timp, Ariel Gershman

**Affiliations:** 1 Department of Biomedical Engineering, Johns Hopkins University, Baltimore, MD, USA; 2 Department of Molecular Biology and Genetics, Johns Hopkins University School of Medicine, Baltimore, MD, USA

The authors present the work “The genome sequence of the lime hawk-moth, Mimas tiliae” where they assemble the genome of the lime hawk-moth. Overall, the genome assembly is very high quality and the authors did a great job presenting the assembly statistics in their figures. This is the first time this species has been assembled, therefore it is obvious that it is beneficial to the field to have such a high quality reference genome. I will note that it’s unfortunate that the authors used a male rather than female as that precludes an assembly of chrW - but in my mind that does not preclude publication in any way.

 My only comments to increase the impact of the study would be to: 

1. In the background section of the paper introduce why the genome of this species is valuable and what biological questions can now be answered. 
2. I would also suggest citation of karotyping and the expectation of the number of chromosomes (which matches the assembly of Traut *et al.* (2007 ^1^)).
3. Utilize the new assembly to answer specific phylogenetic questions by comparing this species to other lepidopterans. 
4. These suggestions are minor and the assembly quality is impressive, I recommend this manuscript for publication. 

Are sufficient details of methods and materials provided to allow replication by others?

Yes

Is the rationale for creating the dataset(s) clearly described?

Yes

Are the datasets clearly presented in a useable and accessible format?

Yes

Are the protocols appropriate and is the work technically sound?

Yes

Reviewer Expertise:

Sequencing technology development

We confirm that we have read this submission and believe that we have an appropriate level of expertise to confirm that it is of an acceptable scientific standard.

## References

[1] : Sex chromosomes and sex determination in Lepidoptera. *Sex Dev* .2007;1(6) : 10.1159/000111765 332-46 10.1159/000111765 18391545

# Peer review: Reviewer response for version 1

**DOI:** 10.21956/wellcomeopenres.19335.r47761

**Published:** 2022-01-05

**Authors:** Ilik J. Saccheri

**Affiliations:** 1 Department of Evolution, Ecology and Behaviour, University of Liverpool, Liverpool, UK

High-quality chromosome-level assembly of a widespread hawk-moth (collected in southern England), including all 29 chromosomes and the mitochondrial genome. This will prove useful for comparative genomics, phylogenetics, and studies of phenotypic variation and adaptation.

- In Fig. 4 it's not clear why only 28, rather than 29, scaffolds are assigned to Arthropoda.
- It would be helpful to state explicitly if the absence of any mention of cobionts represents real absence or omission.

Are sufficient details of methods and materials provided to allow replication by others?

Yes

Is the rationale for creating the dataset(s) clearly described?

Yes

Are the datasets clearly presented in a useable and accessible format?

Yes

Are the protocols appropriate and is the work technically sound?

Yes

Reviewer Expertise:

genomics of adaptation in Lepidoptera

I confirm that I have read this submission and believe that I have an appropriate level of expertise to confirm that it is of an acceptable scientific standard.
